# Dogs as sentinel hosts for sand-fly-borne infections in the Mediterranean Basin: a multinational serological survey

**DOI:** 10.1186/s13071-026-07386-1

**Published:** 2026-04-16

**Authors:** Gaetano Oliva, Gioia Bongiorno, Nazli Ayhan, Iva Kolářová, Valentina Foglia-Manzillo, Oscar David Kirstein, Kristýna Jelínková, Barbora Dvořáková, José Risueño, Elena Verdú-Serrano, Pedro Pérez-Cutillas, José Manuel Cristóvão, M. Magdalena Alcover Amengual, Suha Kenan Arserim, Jesse Barandika Iza, Cristiana Cazapal, Aitor Cevidanes Miranda, Raúl Cuadrado Matías, Sarah Delacour Estrella, Victoriano Díaz-Sáez, Shirly Elbaz, Guillermo Fernández, Roser Fisa, Josefina Garrido, Manuela Gizzarelli, Claudia Fortuna, Aldo Scalone, Claudia Mangiapelo, Ilaria Bernardini, Stefania Orsini, Antonello Amendola, Giulietta Venturi, Trentina Di Muccio, Elif Kurum, Javier Lucientes, Yasmina Martínez, Franjo Martinković, Joaquina Martín-Sánchez, Manuel Morales-Yuste, Yaarit Nachum-Biala, Adolfo Paz Silva, Metin Pekağırbaş, Alejandro Polina, Xavier Roca-Geronès, Francisco Ruiz Fons, Rita Sánchez Andrade, Andrés Torres-Llamas, Kardelen Yetişmiş, Tatjana Živičnjak, Vladimir Ivović, Gad Baneth, Remi Charrel, Yusuf Özbel, Seray Töz, Petr Volf, Carla Maia, Eduardo Berriatua

**Affiliations:** 1 Department of Veterinary Medicine and Animal Production, University of Naples Federico II, Via Federico Delpino 1, 80137 Naples, Italy; 2 Istituto Superiore di Sanità, Viale Regina Elena 299, 00161 Rome, Italy; 3 Present Address: Unité des Virus Émergents (UVE: Aix-Marseille Université, Università di Corsica, IRD 190, Inserm 1207, IRBA), Marseille, France; 4 Department of Parasitology, Faculty of Science, Charles University, Prague, Czech Republic; 5 Public Health Laboratories—Jerusalem (PHL-J), Public Health Services (PHS), Ministry of Health (MOH), Jerusalem, Israel; 6 Departamento de Sanidad Animal, Facultad de Veterinaria, University of Murcia, Campus de Espinardo, Regional Campus of International Excellence ‘Campus Mare Nostrum’, Espinardo, 30100 Murcia, Spain; 7 Departamento de Geografía, Facultad de Letras, University of Murcia, Campus de la Merced, 30001 Murcia, Spain; 8 Global Health and Tropical Medicine (GHTM), Associate Laboratory in Translation and Innovation Towards Global Health, LA-REAL, Instituto de Higiene e Medicina Tropical (IHMT), Universidade Nova de Lisboa (UNL), Lisbon, Portugal; 9 Secció de Parasitologia, Departament de Biologia, Sanitat i Medi Ambient, Facultat de Farmàcia i Ciències de l’Alimentació, Universitat de Barcelona, Barcelona, Spain; 10 Vocational School of Health Sciences, Celal Bayar University, Manisa, Turkey; 11 Basque Institute for Agricultural Research and Development (NEIKER), Derio, Vizcaya Spain; 12 COPAR Research Group, Faculty of Veterinary, University of Santiago de Compostela, 27002 Lugo, Spain; 13 Group in Health and Biotechnology (SaBio), Spanish Game and Wildlife Research Institute—IREC (CSIC-UCLM-JCCM), Ciudad Real, Spain; 14 Animal Health Department, The AgriFood Institute of Aragon (IA2), School of Veterinary Medicine, University of Zaragoza, 50013 Zaragoza, Spain; 15 Departamento de Parasitología, Facultad de Farmacia, University of Granada, Campus Universitario de Cartuja, 18071 Granada, Spain; 16 Quimera Biological Systems, Pol. Malpica-Alfindén C/Olivo 14, Nave 6, La Puebla de Alfinden, 50171 Zaragoza, Spain; 17 Department of Ecology and Animal Biology, Faculty of Biology, University of Vigo, 36310 Vigo, Spain; 18 Faculty of Veterinary Medicine, University of Zagreb, Zagreb, Croatia; 19 School of Veterinary Medicine, The Hebrew University of Jerusalem, Rehovot, Israel; 20 Department of Parasitology, Faculty of Veterinary Medicine, Adnan Menderes University, Aydın, Turkey; 21 CIBERINFEC, National Institute of Health Carlos III (ISCIII), Madrid, Spain; 22 Department of Parasitology, Faculty of Medicine, Ege University, Izmir, Turkey; 23 Faculty of Mathematics, Natural Sciences and Information Technologies, University of Primorska, Koper, Slovenia

**Keywords:** *Leishmania*, Toscana sand fly virus, Sand fly fever Sicilian virus, *Phlebotomus perniciosus* rSP03B salivary protein, *Phlebotomus papatasi* rSP36 salivary protein, Mediterranean countries

## Abstract

**Background:**

Phlebotomine sand-fly-borne infections are an emerging threat to human and animal health in Mediterranean countries, highlighting the need for improved surveillance and control strategies. Dogs are ideal sentinel hosts owing to their central role in the transmission of zoonotic *Leishmania infantum*, frequent exposure to sand fly bites, capacity to develop antibodies to phleboviruses, and close contact with humans. This study reports cross-sectional surveys of antibodies to *Leishmania*, Toscana virus (TOSV), and Sicilian sand fly virus (SFSV) in dogs from Portugal, Spain, Italy, Croatian Istria, Turkey, and Israel, as well as antibodies to salivary proteins of *Phlebotomus perniciosus* (Portugal, Spain, and Italy) and *Phlebotomus papatasi* (Spain and Italy), conducted within the Climate Monitoring and Decision Support Framework for the Detection and Mitigation of Sand fly Diseases with Cost–Benefit and Climate Policy Measures (CLIMOS) project.

**Methods:**

Blood samples and epidemiological data were collected from 2500 dogs. Antibodies to *Leishmania* were detected by indirect immunofluorescence, phlebovirus antibodies by seroneutralization assays, and antibodies to *P. perniciosus* and *P. papatasi* salivary antigens by enzyme-linked immunosorbent assay (ELISA) using recombinant proteins rSP03B and rSP36, respectively. Sources of antibody variability were evaluated using mixed-effects logistic regression models.

**Results:**

Antibodies to *L. infantum*, phleboviruses, and sand fly saliva were widely detected, although seroprevalence varied markedly by region. No *Leishmania*-seropositive dogs were found in Istria, parts of northern Spain, or several districts in Israel, whereas seroprevalence exceeded 30% in Sicily and in several Turkish and Spanish provinces. TOSV seropositivity was generally absent or below 5%, except in southern Spain (8–24%) and Muğla, Turkey (10%). SFSV exposure was highly focal, occurring mainly in Turkey (12%), Israel (12%), and Lisbon (7%). Exposure to *P. perniciosus* was very high in Portugal, Sicily, and most of Spain, while *P. papatasi* exposure was highest in Sicily and selected Spanish regions. Antibody variability was driven primarily by geographical location.

**Conclusions:**

The marked geographical heterogeneity observed confirms dogs as valuable sentinels for sand-fly-borne infections. These infections are highly clustered across Mediterranean regions, likely reflecting differences in sand fly density and infection rates. Understanding the drivers of this heterogeneity is essential for accurate risk mapping and effective control strategies.

**Graphical Abstract:**

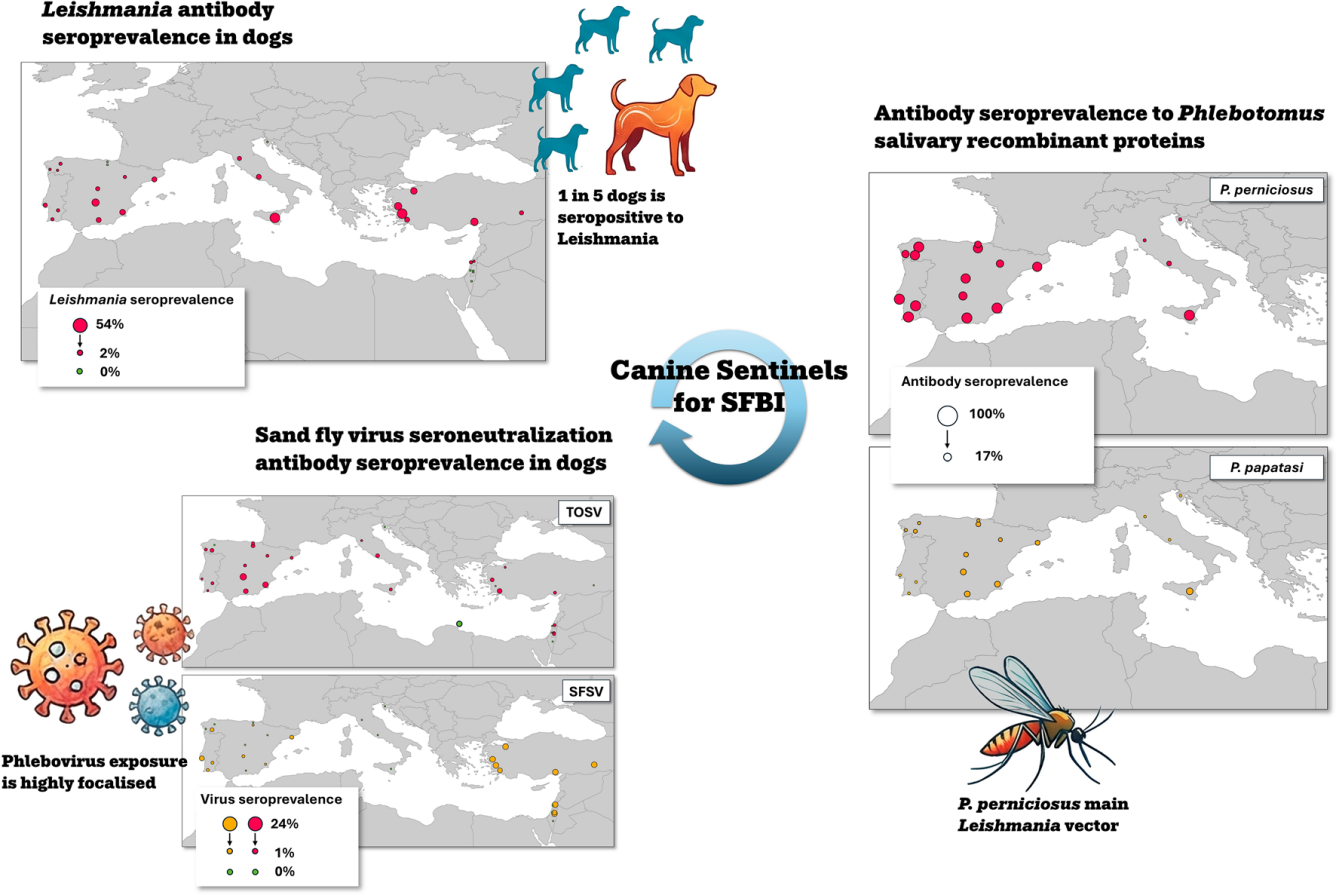

## Background

The significant social role of the dog in urban and rural contexts is demonstrated by the constant growth in their number in Europe, currently estimated at around 90 million [1]. They are potentially an ideal sentinel species owing to their close contact with humans and their susceptibility to sand-fly-borne infections. Their ability to mount measurable antibody immune responses provides a valuable indicator of pathogen circulation, offering critical insights for monitoring, risk assessment, and control of sand-fly-borne diseases in endemic regions. Female phlebotomine sand flies (Diptera: Psychodidae) transmit *Leishmania* protozoan parasites and phleboviruses to humans and animals during blood feeding in warm regions worldwide, including Mediterranean countries. Increasing evidence indicates that the geographic range of sand fly vectors and sand-fly-borne infections (SFBIs) is expanding northward [2–4]. *Leishmania* spp. are major pathogens of humans and animals, and of more than 20 pathogenic species, *Leishmania infantum* is the recognized autochthonous pathogenic species in the European continent [5]. Dogs are the domestic reservoir of *L. infantum* and the most sensitive species, commonly exhibiting a broad spectrum of clinical manifestations, from asymptomatic or subclinical infection to severe, fatal disease known as canine leishmaniosis (CanL) [6]. In dogs, the likelihood of progressing to clinical leishmaniosis depends on the type of immune response that predominates. Effective control of the parasite relies on a Th1-driven cell-mediated response [7]. When this response is weak or absent, a dominant Th2-biased humoral reaction often emerges instead, resulting in excessive production of antibodies. This imbalance can lead to widespread deposition of immune complexes within the capillaries of the skin and various internal organs. Anti-*Leishmania* antibody detection is therefore a useful diagnostic tool of CanL [7]. Controlling CanL and preventing new infections is also epidemiologically important, as both asymptomatic and symptomatic dogs can transmit infection to sand fly vectors [8]. Topical insecticides containing synthetic pyrethroids, which provide both repellent (antifeeding) and insecticidal effects, remain the primary tool for preventing *Leishmania* infection and other sand-fly-borne infections [9].

Phleboviruses represent another group of sand-fly-borne pathogens endemic to southern European countries. Among these, sand fly fever Sicilian virus (SFSV; *Phlebovirus siciliaense*) typically causes a self-limiting febrile illness known as 3-day or sand fly fever, while Toscana virus (TOSV; *Phlebovirus toscanaense*) is of particular concern owing to its neuroinvasive character, which can lead to meningitis or meningoencephalitis in humans [10, 11]. Notably, symptomatic infections of sand-fly-borne phleboviruses in dogs have not been reported. While healthy dogs are unlikely to contribute to the natural transmission cycle of phleboviruses [12], dogs with leishmaniasis may serve as potential amplifying hosts [13] and their seroprevalence can provide useful insights into regional exposure to sand flies and SFBIs. Besides, co-infections with phleboviruses and *Leishmania* have been documented, and experimental mice models indicate that such co-infections may exacerbate leishmaniasis [14].

Serological surveys have detected phlebovirus antibodies in several domestic and wild animals, with neutralizing antibodies against TOSV and SFSV found in up to 8% and nearly 72% in canine population, respectively [15]. These findings underscore the extensive exposure of vertebrate hosts to these viruses.

Exposure to sand fly bites also triggers a robust and specific antibody response against multiple salivary proteins produced by sand fly females and inoculated during the blood feeding [16–18]. Although such markers do not provide a direct assessment of pathogen transmission or infection risk, serological monitoring of antisaliva antibodies is epidemiologically informative, being an indirect, individual measure of the risk for pathogen transmission [19, 20]. In dogs experiencing their first exposure, IgG antibodies to sand fly saliva become detectable in serum within 2–4 weeks, and their levels increase in relation to the number of sand flies that have fed on the host [21, 22]. When the dogs are no longer exposed to sand fly bites, IgG levels decline, making these antibodies a useful indicator of recent sand fly contact [21, 22]. Longitudinal studies in naturally exposed dogs further show that antisaliva antibody levels follow seasonal trends that mirror fluctuations in sand fly presence [19, 20, 23]. To date, enzyme-linked immunosorbent assays (ELISAs) have been developed to detect antibodies against the saliva of the sand fly species *P. perniciosus* and *P. papatasi* in dogs, using as antigens either crude salivary gland homogenates (SGH) or recombinant proteins such as PER-rSP03B and PAP-rSP36, respectively [24–26]. These recombinant proteins offer clear advantages over the use of SGH, as they can be produced on a large scale, eliminating the need for the labor-intensive dissection of salivary glands from numerous specimens [27]. *Phlebotomus perniciosus* is widespread and the main *L. infantum* vector in Western Europe, including Portugal, Spain, and Italy [28]. *Phlebotomus papatasi* is also widespread in these countries, but its vectorial capacity is restricted to *Leishmania major* [29], a species endemic in Northern Africa, the Middle East, and Turkey [28]. *Phlebotomus perniciosus* is also a competent vector for TOSV, while *P. papatasi* is refractory to it [30], being traditionally associated with “Papatasi fever,” a human illness caused by sand fly fever Sicilian virus (SFSV) and other phleboviruses [31].

The role of sand flies as emerging vectors of zoonotic pathogens has led to increasing interest in the systematic monitoring of SFBIs and in identifying sand fly presence in new geographical areas. The present study reports the results of a 2-year cross-sectional investigation of the prevalence of antibodies against *Leishmania*, TOSV, SFSV, and *P. perniciosus* and *P. papatasi* salivary antigens in dogs in six Mediterranean countries: Portugal, Spain, Italy, Croatia, Turkey, and Israel. The study is part of the European project CLIMOS, Climate Monitoring and Decision Support Framework for the Detection and Mitigation of Sand fly Diseases with Cost–Benefit and Climate Policy Measures (CLIMOS: http://www.climosproject.eu), which aims to mitigate the impact of climate change on the spread of vector-borne and zoonotic diseases, by applying Eco-health and One Health approaches, investigating how climate and environmental factors affect sand fly populations and the diseases they transmit across Europe.

## Methods

### Study population and study design

**Fig. 1:**
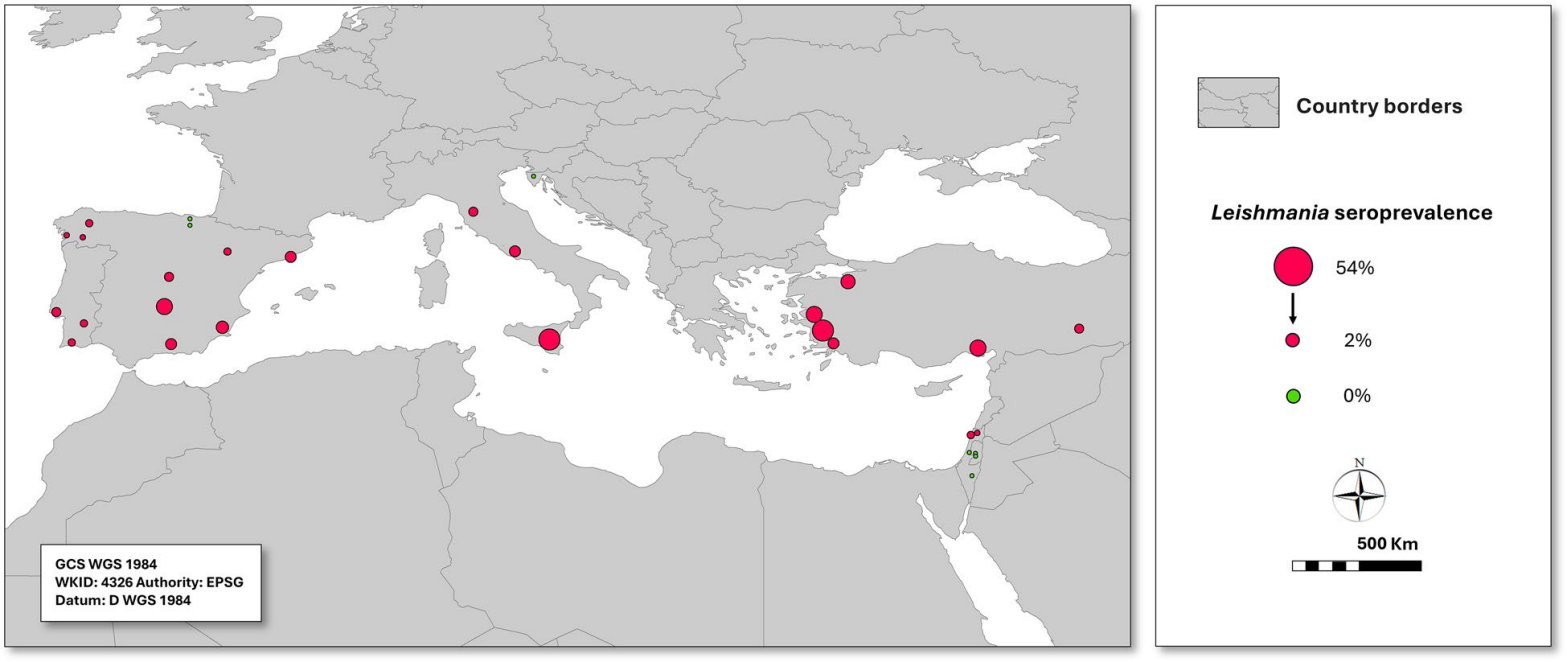
*Leishmania infantum* IFAT antibody seroprevalence in dogs in selected areas of Portugal, Spain, Italy, Croatia, Turkey, and Israel. Values are presented on a continuous scale; the largest and smallest circles correspond to the maximum and minimum values shown in the legend

The study was conducted in dogs residing near CLIMOS sand fly surveillance sites in Portugal, Spain, Italy, Croatia, Turkey, and Israel, covering 30 Nomenclature of Territorial Units for Statistics, Level 3 (NUTS3) areas in Europe and Turkey, as well as six districts in Israel (Fig. 1; Tables 1, 2, 3). Sample sizes were estimated at 60 dogs per NUTS2 region (including one or more NUTS3 and districts), to allow detecting at least one seropositive dog with a 95% probability, assuming a conservative regional seroprevalence of 5%.

**Table 1:** Seroprevalence of antibodies to *Leishmania infantum*, Toscana sand fly virus (TOSV), and sand fly fever Sicilian virus (SFSV) according to explanatory variables

Variable	Level	No. of dogs	% seropositive (95% confidence limits)		
			*L. infantum*	TOSV	SFSV
All		2204–2401	18 (16–19)	5 (4–6)	5 (4–6)
Country	Croatia	27	0 (0–0)*	0 (0–0)*	0 (0–0)*
	Israel	172–199	2 (0–3)	2 (0–4)	12 (7–16)
	Italy	357	28 (24–33)	3 (1–4)	0 (0–0)
	Portugal	349–351	9 (6–12)	1 (0–3)	4 (2–6)
	Spain	806–976	15 (13–17)	9 (7–11)	1 (0–2)
	Turkey	488–491	29 (25–33)	4 (2–5)	12 (9–15)
NUTS3/district	Croatia, Istria	27	0 (0–0)*	0 (0–0)*	0 (0–0)*
	Israel, Central	0–1	0 (0–0)	0 (0–0)	0 (0–0)
	Israel, Haifa	9–13	8 (0–22)	0 (0–0)	0 (0–0)
	Israel, Jerusalem	37–38	0 (0–0)	3 (0–8)	14 (2–25)
	Israel, Judea, Samaria	28–33	0 (0–0)	3 (0–10)	7 ( 0–17)
	Israel, Northern	85–100	2 (0–5)	2 (0–5)	15 (8–23)
	Israel, Southern	13–14	0 (0–0)	0 (0–0)	0 (0–0)
	Italy, Lazio	105	18 (11–25)	4 (0–7)	0 (0–0)
	Italy, Sicily	117	54 (45–63)	3 (0–7)	0 (0–0)
	Italy, Tuscany	135	14 (8–20)	1 (0–2)	0 (0–0)
	Portugal, Alentejo	73–74	8 (2–14)	3 (0–6)	3 (0–6)
	Portugal, Algarve	153	8 (4–12)	1 (0–3)	2 (0–4)
	Portugal, Lisbon	123–124	11 (6–17)	1 (0–2)	7 (3–12)
	Spain, Araba	85–82	0 (0–0)	5 (0–9)	1 (0–3)
	Spain, Barcelona	76–117	15 (9–22)	3 (0–6)	4 (0–8)
	Spain, Bizkaia	32–40	0 (0–0)	3 (0–9)	0 (0–0)
	Spain, Ciudad, Real	135–145	34 (27–42)	24 (17–32)	2 (0–5)
	Spain, Granada	86–121	18 (11–25)	8 (2–14)	0 (0–0)
	Spain, Lugo	19–42	7 (0–15)	0 (0–0)	0 (0–0)
	Spain, Madrid	107–109	11 (5–17)	2 (0–4)	0 (0–0)
	Spain, Murcia	133–147	24 (17–31)	16 (10–22)	1 (0–3)
	Spain, Ourense	25–30	3 (0–10)	4 (0–12)	4 (0–11)
	Spain, Pontevedra	35–40	3 (0–7)	3 (0–8)	0 (0–0)
	Spain, Zaragoza	70–103	6 (1–10)	3 (0–7)	0 (0–0)
	Turkey, Adana	83–80	31 (21–41)	2 (0–6)	16 (8–23)
	Turkey, Aydin	56	54 (41–67)	0 (0–0)	13 (4–21)
	Turkey, Bursa	78–71	27 (16–37)	1 (0–4)	15 (7–23)
	Turkey, Diyarbakir	50–49	10 (2–19)	0 (0–0)	14 (4–24)
	Turkey, Manisa	112–158	33 (26–40)	4 (0–7)	13 (6–19)
	Turkey, Mugla	109–77	17 (9–25)	10 (4–16)	6 (1–10)
Country (year)	Croatia, 2023–24	27	0 (0–0)	0 (0–0)*	0 (0–0)*
	Israel, 2022–23	176–199	2 (0–3)	2 (0–4)	12 (7–16)
	Italy, 2023–24	102	47 (37–57)	2 (0–5)	0 (0–0)
	Italy, 2024–25	255–255	21 (16–26)	3 (1–5)	0 (0–0)
	Portugal, 2023–24	140	14 (8–19)	1 (0–2)	4 (0–7)
	Portugal, 2024–25	209–211	6 (3–9)	2 (0–4)	4 (2–7)
	Spain, 2023–24	339–444	14 (11–17)	15 (12–19)	0 (0–1)
	Spain, 2024–25	467–532	16 (13–20)	5 (3–7)	2 (1–3)
	Turkey, 2023–24	488–491	29 (25–33)	4 (2–5)	12 (9–15)
Season	January–March	632–646	11 (9–13)*	3 (2–5)*	4 (2–5)*
	April–June	446–452	21 (17–25)	2 (1–4)	3 (2–5)
	July–September	209–227	12 (8–16)	1 (0–3)	13 (9–18)
	October–December	912–1073	22 (19–24)	8 (6–10)	4 (3–5)
Sex	Female	966–1078	17 (15–19)	6 (4–7)	5 (3–6)
	Male	1162–1242	19 (17–22)	5 (3–6)	4 (3–5)
Age (years)	0.5–3.0	831–902	16 (14–18)*	3 (2–5)*	6 (4–7)
	3.1–7.0	710–769	21 (18–23)	5 (4–7)	5 (3–6)
	7.1–18	591–661	17 (14–19)	7 (5–9)	3 (2–5)
Breed	Mixed	1216–1287	23 (21–25)*	4 (3–5)*	7 (6–8)*
	Pure autochthonous	402–423	13 (10–16)	7 (4–9)	1 (0–2)
	Pure non-autochthonous	506–607	11 (9–14)	6 (4–8)	2 (1–3)
Hair length	Long	141–174	13 (8–18)	9 (4–13)	0 (0–0)*
	Medium	251–303	14 (10–18)	5 (2–7)	1 (0–2)
	Short	709–774	16 (14–19)	6 (5–8)	5 (4–7)
Type of residence	Farmstead	79	18 (9–26)*	23 (14–32)*	4 (0–8)*
	Hunting kennel	427–434	12 (9–15)	2 (1–4)	1 (0–1)
	Private household	724–853	16 (14–19)	6 (4–7)	4 (2–5)
	Shelter kennel	950–1003	22 (20–25)	4 (3–6)	7 (5–9)
Living environment	Indoors	237–301	11 (7–14)*	4 (2–7)	1 (0–2)*
	Indoors and outdoors	1844–2070	19 (17–21)	5 (40–6)	5 (4–6)
Open-air at night	No	416–503	11 (9–14)*	8 (5–11)*	1 (0–2)*
	Yes	1619–1707	21 (19–23)	4 (3–5)	5 (4–6)
Ectoparasiticides	No	774–799	24 (21–27)*	6 (4–8)	9 (7–10)*
	Yes	1311–1462	16 (14–18)	5 (3–6)	2 (1–3)
SF^a^ collar	No	1671–1763	19 (18–21)*	5 (4–6)	5 (4–6)
	Yes	296–362	14 (10–17)	5 (3–8)	2 (1–4)
SF^a^ pipettes	No	1675–1776	17 (15–19)*	5 (4–6)	5 (4–6)*
	Yes	269–302	28 (23–34)	5 (2–7)	1 (0–2)
SF^a^ collar or pipette	No	1398–1453	18 (16–20)*	5 (4–6)	6 (4–7)*
	Yes	538–616	21 (18–25)	5 (3–7)	2 (1–3)
SF^a^ collar and pipette	No–No	1398–1453	18 (16–20)*	5 (4–6)	6 (4–7)*
	No–Yes	239–243	31 (25–37)	5 (2–7)	0 (0–1)
	Yes–No	261–302	14 (10–18)	5 (2–7)	3 (1–5)
	Yes–Yes	27–48	8 (1–16)	7 (0–17)	0 (0–0)
Leish.^b^ vaccination	No	2070–2246	18 (16–19)	5 (4–6)	5 (4–6)
	Yes	116–133	22 (15–29)	5 (1–9)	2 (-1–4)
Any clinical signs	No	1547–1137	19 (16–21)	6 (5–7)	5 (4–6)
	Yes	236–151	25 (18–31)	5 (2–7)	7 (4–10)
Leish.^b^ clinical signs	No	1601–1706	19 (17–21)*	6 (5–7)	5 (4–6)
	Yes	158–271	27 (21–32)	5 (2–8)	6 (3–10)

* *P* < 0.05

^a^Sand fly repellent/insecticide

^b^*Leishmania infantum*

**Table 2:** Results from the mixed-effects logistic regression analysis of dog’s antibody seropositivity to *L. infantum*, TOSV, SFSV, *P. perniciosus* rSPO3B, and *P. papatasi* rSP36

Variable	Level	Odds ratio	95% confidence limits		*P* value
*L. infantum*					
High night exposure to sand flies	No	1.00			
	Yes	2.38	1.49	3.79	0.0003
Preventive ectoparasiticides	No	1.00			
	Yes	0.47	0.25	0.89	0.0198
Sand fly repellents/insecticides	No	1.00			
	Yes	2.03	1.14	3.61	0.0164
*L. infantum* vaccination	No	1.00			
	Yes	2.14	1.29	3.57	0.0016
*L. infantum* clinical signs	No	1.00			
	Yes	3.30	2.17	5.02	0.0000
NUTS3/district: variance estimate	0.818				
TOSV					
Age	0.5–3.0	1.00	1.00	1.00	
	3.1–7.0	1.64	0.95	2.81	0.0751
	7.1–18	2.27	1.31	3.92	0.0033
Breed	Mixed	1.00	1.00	1.00	
	Pure autochthonous	1.71	0.92	3.18	0.0880
	Pure non-autochthonous	1.29	0.74	2.28	0.3712
NUTS3/district: variance estimate	0.9039				
SFSV					
Residence	Shelter kennel	1.00	1.00	1.00	
	Farmstead	1.50	0.26	8.60	0.650
	Hunting kennel	0.20	0.04	1.15	0.072
	Private household	0.85	0.50	1.46	0.563
	0.8622				
*P. perniciosus* rSPO3B					
Residence	Shelter kennel	1.00	1.00	1.00	
	Farmstead	0.10	0.03	0.30	0.000
	Hunting kennel	0.10	0.04	0.22	0.000
	Private household	0.28	0.14	0.58	0.001
SF repellents/insecticides	No	1.00	1.00	1.00	
	Yes	0.59	0.35	0.98	0.041
NUTS3/district: variance estimate	3.6672				
*P. papatasi* rSP36					
Age	0.5–3.0	1.00	1.00	1.00	
	3.1–7.0	2.02	1.43	2.85	0.000
	7.1–18	1.61	1.15	2.26	0.006
High night exposure to SF	No	1.00	1.00	1.00	
	Yes	1.44	1.06	1.94	0.018
NUTS3/district: variance estimate	0.2121

**Table 3:** Antibody seroprevalence (95% confidence limits) and median (interquartile range: IQ) standardized optical densities against *Phlebotomus perniciosus* PER-rSP03B and *Phlebotomus papatasi* PAP-rSP36 salivary recombinant proteins, according to explanatory variables

Variable	Level	No. dogs	*P. perniciosus*		*P. papatasi*	
			Seroprevalence	Median (IQ)	Seroprevalence	Median (IQ)
All		1087–1540	84 (82–86)*	27 (17–40)	36 (33–39)	24 (19–30)
Country	Italy	114–217	68 (62–74)	41 (25–65)*	57 (48–66)*	25 (18–35)*
	Portugal	351	100 (100–100)	34 (24–48)	–	–
	Spain	972–973	82 (80–85)	23 (14–34)	34 (31–37)	24 (20–29)
Region/province	Italy, Lazio	103	37 (28–46)*	27 (23–36)*	–	–
	Italy, Sicily	114	96 (93–100)	53 (31–69)	57 (48–66)	25 (18–35)
	Italy, Tuscany	0	–	–	–	–
	Portugal, Alentejo	74	100 (100–100)	42 (31–59)	–	-
	Portugal, Algarve	153	100 (100–100)	27 (20–39)	–	–
	Portugal, Lisbon	124	100 (100–100)	37 (27–49)	–	–
	Spain, Araba	86	83 (75–91)	21 (14–27)	36 (26–46)	22 (18–26)
	Spain, Barcelona	116	82 (75–89)	26 (15–37)	31 (23–39)	25 (21–28)
	Spain, Bizkaia	40	58 (42–73)	14 (11–15)	18 (6–29)	21 (20–21)
	Spain, Ciudad, Real	142	70 (63–78)	23 (14–37)	41 (33–49)	25 (20–30)
	Spain, Granada	122	95 (91–99)	24 (12–34)	43 (35–52)	24 (19–30)
	Spain, Lugo	42	95 (89–102)	21 (13–28)	17 (5–28)	23 (20–36)
	Spain, Madrid	109	88 (82–94)	18 (9–30)	32 (23–41)	25 (20–30)
	Spain, Murcia	146	92 (88–97)	27 (17–37)	41 (33–49)	23 (20–27)
	Spain, Ourense	30	90 (79–101)	23 (19–27)	23 (8–38)	24 (22–29)
	Spain, Pontevedra	39–40	67 (52–81)	25 (17–37)	20 (8–32)	26 (22–29)
	Spain, Zaragoza	100	70 (61–79)	25 (15–34)	28 (19–37)	25 (20–30)
Year	Italy, 2023, 24	58–97	90 (84–96)*	41 (25–62)*	62 (50–75)*	35 (27–42)*
	Italy, 2024, 25	56–120	51 (42–60)	43 (25–68)	52 (39–65)	17 (13–21)
	Portugal, 2023, 24	140	100 (100–100)	30 (23–40)	–	–
	Portugal, 2024, 25	211	100 (100–100)	37 (25–53)	–	–
	Spain, 2023, 24	441–442	64 (59–68)	29 (21–37)	25 (21–29)	25 (21–31)
	Spain, 2024, 25	531	97 (96–99)	18 (12–29)	41 (37–45)	23 (19–28)
Season	01, Jan–Mar	256–537	97 (95–98)*	26 (16–39)*	30 (25–36)*	22 (18–27)*
	02, Apr–June	56–190	69 (62–76)	39 (26–60)	52 (39–65)	17 (13–21)
	03, July, Sep	5	40 (− 3 to 83)	35 (34–36)	0 (0–0)	0 (0–0)
	04, Oct, Dec	767–805	80 (77–83)	26 (15–38)	37 (34–41)	25 (20–31)
Sex	Female	512–713	85 (82–87)	25 (15–38)*	34 (30–38)	24 (19–30)*
	Male	556–807	84 (82–87)	29 (18–42)	39 (35–43)	23 (20–30)
Age (years)	0.5–3.0	301–470	81 (78–85)*	27 (17–37)	28 (23–33)*	26 (20–31)*
	3.1–7.0	346–506	89 (86–91)	28 (17–44)	45 (40–50)	23 (19–30)
	7.1–18	427–546	83 (80–86)	26 (16–38)	36 (31–40)	23 (19–28)
Breed	Mixed	458–693	89 (86–91)*	29 (19–45)*	42 (38–47)*	23 (19–30)*
	Pure autochthonous	137–317	76 (71–81)	28 (18–41)	39 (31–48)	26 (22–32)
	Pure non-autochthonous	444–479	83 (80–86)	23 (14–35)	30 (26–34)	23 (19–29)
Hair length	Long	169	80 (74–86)	22 (14–34)*	33 (25–40)	23 (19–28)*
	Medium	239	81 (76–86)	20 (13–31)	31 (25–37)	23 (19–28)
	Short	441–501	75 (71–78)	25 (14–34)	33 (29–37)	24 (20–29)
Type of residence	Farmstead	61–77	71 (61–82)*	23 (15–35)*	39 (27–52)*	23 (21–26)*
	Hunting kennel	42–297	82 (78–87)	30 (23–44)	43 (28–58)	23 (21–27)
	Private household	594–593	83 (80–86)	23 (13–34)	31 (27–35)	23 (19–29)
	Shelter kennel	377–560	89 (86–91)	31 (20–48)	44 (38–49)	24 (19–32)
Living environment	Indoors	298	82 (77–86)	20 (11–32)	32 (26–37)*	24 (19–29)*
	Indoors and outdoors	317–846	85 (83–87)	29 (19–43)	38 (35–42)	23 (19–30)
Night outside	No	497	82 (79–85)	20 (11–32)*	31 (27–35)*	24 (20–29)*
	Yes	544–997	86 (84–88)	29 (18–42)	42 (38–46)	23 (19–30)
Ectoparasiticides^1^	No	175–264	89 (86–93)*	23 (14–34)*	41 (33–48)	26 (20–30)*
	Yes	895–1257	83 (81–85)	30 (19–44)	35 (32–39)	23 (19–30)
SF collar	No	692–1049	86 (84–89)	25 (17–39)*	39 (35–42)*	24 (19–30)*
	Yes	309–357	83 (79–87)	27 (16–40)	32 (27–37)	23 (19–30)
Sf pipettes	No	680–1056	85 (83–87)	26 (16–39)*	36 (32–39)	23 (19–29)*
	Yes	272–301	87 (83–91)	32 (19–51)	37 (31–42)	25 (19–33)
SF collar or pipette	No	411–739	85 (82–87)	26 (16–39)*	36 (32–41)	23 (19–29)*
	Yes	534–611	86 (83–89)	29 (17–41)	35 (31–39)	24 (19–31)
SF collar and pipette	No–No	411–739	85 (82–87)*	26 (16–39)*	36 (32–41)*	23 (19–29)*
	No–Yes	214–243	91 (87–94)	36 (20–55)	41 (35–48)	25 (19–34)
	Yes–No	250–298	86 (82–90)	27 (16–38)	34 (28–39)	23 (19–28)
	Yes–Yes	47	68 (55–81)	26 (14–33)	19 (8–30)	24 (19–29)
Leish^2^. vaccination	No	944–1386	84 (83–86)	27 (17–41)*	37 (34–40)	24 (20–30)*
	Yes	130–132	85 (79–91)	23 (14–35)	33 (25–41)	23 (18–29)
Clinical signs	No	906–993	81 (79–84)	25 (14–37)*	38 (35–41)	24 (19–30)*
	Yes	136–151	77 (70–84)	24 (15–35)	29 (22–37)	25 (20–31)
Leish^2^. clinical signs	No	961–1049	82 (79–84)	25 (15–37)*	38 (35–41)	24 (19–30)*
	Yes	76–90	72 (63–81)	23 (14–36)	34 (24–45)	24 (20–32)

^1^Sand fly repellent/insecticide

^2^*Leishmania infantum*

* *P* < 0.05

Blood samples and individual epidemiological questionnaires were collected, with owners/guardian’s informed consent, from 2500 dogs. Sample sizes varied widely between countries, ranging from 993 dogs in Spain and 27 dogs in Croatia (Tables 1–3). The majority of samples (78%) were taken in the months after the main annual sand fly season, between October and April, in 2023–24 and in 2024–25, but this was not always possible and collection periods varied by country: Israel (Mar 2022–Aug 2023), Croatia (Mar–June 2023), Italy (Oct 2023–May 2025), Portugal (Mar 2024–Apr 2025), Spain (Sep 2023–Mar 2025), and Turkey (July 2023–Apr 2024). The dogs originated from shelters (*n* = 1037 dogs) in every country, private households (*n* = 918) in Israel, Spain, and Turkey, hunting kennels (*n* = 434) in Italy, Portugal, and Spain, and farms (*n* = 79) in Portugal and Spain. All dogs underwent a physical examination to rule out any clinical conditions requiring immediate treatment. Lactating and pregnant dogs were excluded.

Serum was obtained from clotted blood samples and tested for antibodies against *Leishmania* spp., Toscana virus (TOSV), and sand fly fever Sicilian virus (SFSV) in all participating countries. In addition, sera from Italy and Spain were tested for antibodies against *Phlebotomus perniciosus* and *P. papatasi* sand fly saliva antigens, while samples from Portugal were tested only for *P. perniciosus*. Because of limited serum volumes, antibody testing was not available for all dogs; sample sizes ranged from 2204 to 2401 for *Leishmania* spp. and phleboviruses (Table 1) and from 1087 to 1540 for salivary antigens (Table 3).

### Indirect immunofluorescence *Leishmania* antibody testing

Detection of anti-*Leishmania* IgG antibodies was performed by an in-house indirect immunofluorescence antibody test (IFAT). *Leishmania infantum* promastigotes (WHO reference strain MHOM/TN/1980/IPT-1) were used as antigen and coated in multispot slides (BIO-RAD, #50576). After air drying, promastigotes were fixed in cold acetone. Sera samples were serially diluted in phosphate-buffered saline (PBS) from 1/40 to 1/5120. Thirty microliters of each dilution were applied to individual wells of the multispot slides and incubated for 30 min at 37 °C. Slides were then washed with PBS and incubated for 30 min at 37 °C with fluorescein isothiocyanate (FITC)-conjugated anti-dog IgG (Sigma, #F4012) diluted 1:200 in PBS. After a further washing step in PBS, slides were mounted with a coverslip using PBS/glycerol (50% v/v) and examined under a fluorescent microscope at 40× magnification using a FITC filter. Each slide included both positive and negative control sera. The antibody titer was defined as the highest serum dilution showing specific fluorescence of *Leishmania* promastigotes. The ≥ 1:80 cutoff was selected in accordance with commonly applied thresholds in epidemiological surveys and to maximize sensitivity for detecting exposure in asymptomatic dogs [32].

### Seroneutralization assay for phlebovirus antibody testing

Serum samples were heat-inactivated at 56 °C for 30 min and subjected to four serial twofold dilutions in 96-well plates using an epMotion 5075 liquid-handling system (Eppendorf, Hamburg, Germany). Each dilution was incubated for 1 h at 37 °C with 100 TCID50 of either TOSV (MRS2014-44725) or (Sabin) to allow antibody–virus interaction. Subsequently, 100 µL of Vero E6 cell suspension (5 × 10^5^ cells/mL) was added to each well using the epMotion 5075 system. Negative and positive controls were included on every plate. Plates were monitored using the Incucyte SX5 Live-Cell Analysis System (Sartorius, Göttingen, Germany), which captured images of each well in the 96-well microplates. Cytopathic effect (CPE) and neutralization titers were evaluated on days 5 and 6 post-infection for TOSV and SFSV, respectively. Samples with neutralization titers ≥ 40 were considered positive. All procedures except serum dilutions were conducted under biosafety level 3 (BSL-3) conditions.

### Enzyme-linked immunosorbent assay (ELISA) for detection of antibodies against *Phlebotomus* spp. salivary antigens

The level of anti-sand fly saliva IgG antibodies in dogs was used to estimate exposure to sand flies. Recombinant protein-based ELISA was used to measure exposure to *P. perniciosus* and *P. papatasi* bites. Two recombinant salivary proteins (rSP) were used as the antigen, either *P. perniciosus* rSP03B (PER-rSP03B) or *P. papatasi* rSP36 (PAP-rSP36), both expressed in *Escherichia coli* as described by Drahota et al. [24] and Kolarova et al. [26], respectively. The ELISAs were performed in three different laboratories following the same standardized protocol as follows. The lyophilized recombinant protein was reconstituted to the original volume with distilled water and diluted to 4 µg/ml in 20 mM carbonate-bicarbonate buffer (pH 9.6). ELISA plates (Thermo Scientific, #3855) were coated with 50 µl/well (i.e., 0.2 µg/well) for 2 h at room temperature (RT). After washing with PBS-Tween (1× PBS with 0.05% Tween 20), the plates were blocked with 6% non-fat dried milk (ITW Reagents, #A0830) diluted in PBS-Tween and incubated for 60 min at 37 °C. After another washing step, the plates were incubated with sera diluted 1:300 in 2% nonfat dried milk for 60 min at room temperature (RT), followed by an overnight incubation at 4 °C. The next day, the plates were washed and incubated for 60 min at 37 °C with peroxidase-conjugated anti-dog IgG (Bethyl Laboratories, #A40-123P), diluted 1:40,000 in PBS-Tw. The chromogenic reaction was developed using a TMB substrate solution (3,3′,5,5′-tetramethylbenzidine, Sigma-Aldrich, #T4444) in the dark for 10 min, after which it was stopped by the addition of 10% sulfuric acid. Absorbance was measured at 450 nm. Each serum sample was tested in duplicate, and each plate contained a set of control sera: a positive control (PC), a cut-off control (C/O), and a negative control (NC). Positive and C/O control sera originated from the naturally bitten Spanish (*P. perniciosus* PC) and Turkish dogs (*P. papatasi* PC) sampled at the end of sand fly season and evaluated in preliminary experiments. Negative control sera originated from the Czech Republic, a sand-fly-free area. Cutoff was calculated as mean optical density of sera from the Czech Republic plus three standard deviations; Spanish (*P. perniciosus* C/O) and Turkish (*P. papatasi* C/O) canine sera showing an optical density at the cutoff value were selected as C/O controls. Dogs showing the antibody level above the cut-off were considered seropositive.

### Statistical analysis

Sand fly salivary antibody optical densities were standardized using the following formula SOD = (S_OD_ − NC_OD_)/(PC_OD_ − NC_OD_), with S, NC, and PC representing the sample and the negative and positive controls, respectively [33]. The mean SOD was then calculated for each sample. Mean negative SOD values were set to zero, normalized using a decimal logarithmic transformation of SOD + 1, and multiplied by 100 to obtain LOD values. Seroprevalence was defined as the proportion of dogs testing antibody-positive and used to calculate confidence intervals and they were presented as percentages. Median LODs and proportion of antibody-positives were compared across explanatory variables using non-parametric Kruskal–Wallis and chi-squared (or when required, Fisher exact) test, respectively.

Given the hierarchical structure of the data and the potential for spatial clustering of *Leishmania* infection, mixed-effects logistic regression models were used to assess the relationship between seropositivity (binary outcome) and explanatory variables associated with the outcome for *P* < 0.10 in the bivariate analysis and including province (NUTS3/district) as a random intercept [34]. Fixed effects were sex, age, breed, type of residence, outdoor access, night exposure to sand flies (stay outdoors or in open-air premises), ectoparasiticidal prophylactic treatments (licensed for sand flies or not), *Leishmania* vaccination with LetiFend^®^ (Leti Pharma), clinical signs (any), and *Leishmania*-compatible clinical signs according to Solano-Gallego et al. [6].

To quantify variance partitioning and model explanatory power, intraclass correlation coefficients (ICC) and *R*^2^ statistics [35] were employed. The unadjusted and adjusted ICCs were used to estimate the proportion of total variance attributable to differences between provinces before and after adjustment for fixed effects. In addition, marginal *R*^2^ (reflecting variance explained by fixed effects alone) and conditional *R*^2^ (reflecting variance explained by both fixed and random effects) were computed to assess the relative contributions of measured covariates and unmeasured province-level factors. These complementary metrics were used to justify the inclusion of province as a random effect and to evaluate residual spatial heterogeneity in *Leishmania* infection risk.

Modeling followed a backward elimination approach, beginning with a saturated model that included all explanatory variables. Diagnostic tests for overdispersion, zero inflation, and collinearity—assessed using the variance inflation factor (VIF)—were performed [36]. The final models included variables significantly (*P* < 0.05) or marginally significantly (*P* < 0.10) associated with the outcome in two-sided tests, and odds ratios (ORs) were used as the measure of association. All analyses were conducted in R [37]. Mixed-effects models were fitted using the lme4 package, and ICC and *R*^2^ estimates were derived using the performance and MuMIn packages.

## Results

### Seroprevalence of *Leishmania* and relationships with explanatory variables

The estimated overall *Leishmania* IFAT seroprevalence among 2401 dogs analyzed was 18% (95% CI: 16–19%), with country-level estimates in decreasing order, of 29% (25–33%) in Turkey, 28% (24–33%) in Italy, 15% (13–17%) in Spain, 9% (6–12%) in Portugal, 2% (0–3%) in Israel, and 0% in Croatia (Table 1; Fig. 1). Seroprevalence varied significantly among regions within Turkey, Italy, and Spain (*P* < 0.05). In Turkey, seroprevalence ranged from 10% in Diyarbakir to 54% in Aydin. In Italy, it was 54% in Sicily, 18% in Lazio, and 14% in Tuscany. In Spain, it was lowest in the northern regions of Araba and Bizkaia (0%) and Ourense and Pontevedra (3%), and highest in the southern provinces of Ciudad Real (34%) and Murcia (24%) (*P* < 0.05) (Table 1; Fig. 1). Seroprevalence was greater in 2023–24 than in 2024–25 in Italy and Portugal, which was related to significant increases in seroprevalence in Sicily and Setubal in the Lisbon Metropolitan Region (M.R.) (*P* < 0.05). Also, there were significant seasonal differences in seroprevalence, with 21–22% in Apr–June and Oct–Dec and with 11–12% in Jan–Mar and July–Sep (Table 1).

On the bivariate statistical analysis, seroprevalence differed significantly according to dog’s age, breed, type of residence, use of ectoparasiticides, and presence of clinical signs compatible with CanL (*P* < 0.05) (Table 1). Seroprevalence rose from 16% among 0.5–3-year-olds to 21% in 3.1–7.0-year-olds, declining to 16% in older dogs. It was 23% in mixed breed dogs and 13% and 11% in autochthonous pure and non-autochthonous breeds, respectively. Depending on the dog’s residence, seroprevalence ranged from 22% in dogs in kennel shelters and 12% in those from hunting kennels, and was 18% in dogs in farms and 16% in those from private households. Also, seroprevalence was highest in dogs living permanently outdoors (19%) or with night exposure to sand flies (21%) compared with other dogs. Regarding preventive ectoparasiticide treatments, seroprevalence was 16% in treated compared with 24% among untreated dogs. However, among dogs using licensed anti-sand-fly topical products (collars, pipettes, or both), seroprevalence was 14% in those using collars only, 31% in those using pipettes only, and 8% in those using both, compared with 18% in untreated dogs. Finally, seroprevalence was 27% in dogs showing clinical signs compatible with CanL versus 19% in those considered clinically healthy (*P* < 0.05) (Table 1).

The distribution of antibody titers among 413 seropositive dogs were 1/80 (198 dogs), 1/160 (95 dogs), 1/320 (71 dogs), 1/640 (26 dogs), 1/1280 (15 dogs), and 1/2560 (8 dogs). The proportion of dogs with high IFAT antibody titers (≥ 1:320) varied between countries, being highest in Italy and Spain compared with Portugal and Turkey; it was lowest in dogs sampled in January–March, being higher in dogs receiving ectoparasiticidal treatments and dogs with clinical signs (*P* < 0.05).

Saturated logistic regression models showed no evidence of overdispersion or zero inflation. The variable describing the dog’s housing type (kennel, farm, or private households) showed strong collinearity with other explanatory variables and was therefore excluded from further analyses. Following stepwise removal of variables not associated with the outcome, the final model identified positive associations between seropositivity and night-time exposure to sand flies, use of collars or pipettes licensed for sand flies, *Leishmania* vaccination, and the presence of clinical signs compatible with canine leishmaniosis. In contrast, seropositivity was negatively associated with the use of ectoparasiticides (any). However, the model indicated strong spatial clustering of infection, with most of the explained variability associated to the random effect and not by the fixed effects (conditional *R*^2^ = 0.264; marginal *R*^2^ = 0.081). This was further supported by an intraclass correlation coefficient (adjusted ICC: 0.20; unadjusted ICC: 0.18) (Table 2).

### Seroprevalence of TOSV and SFSV and relationships with explanatory variables

**Fig. 2:**
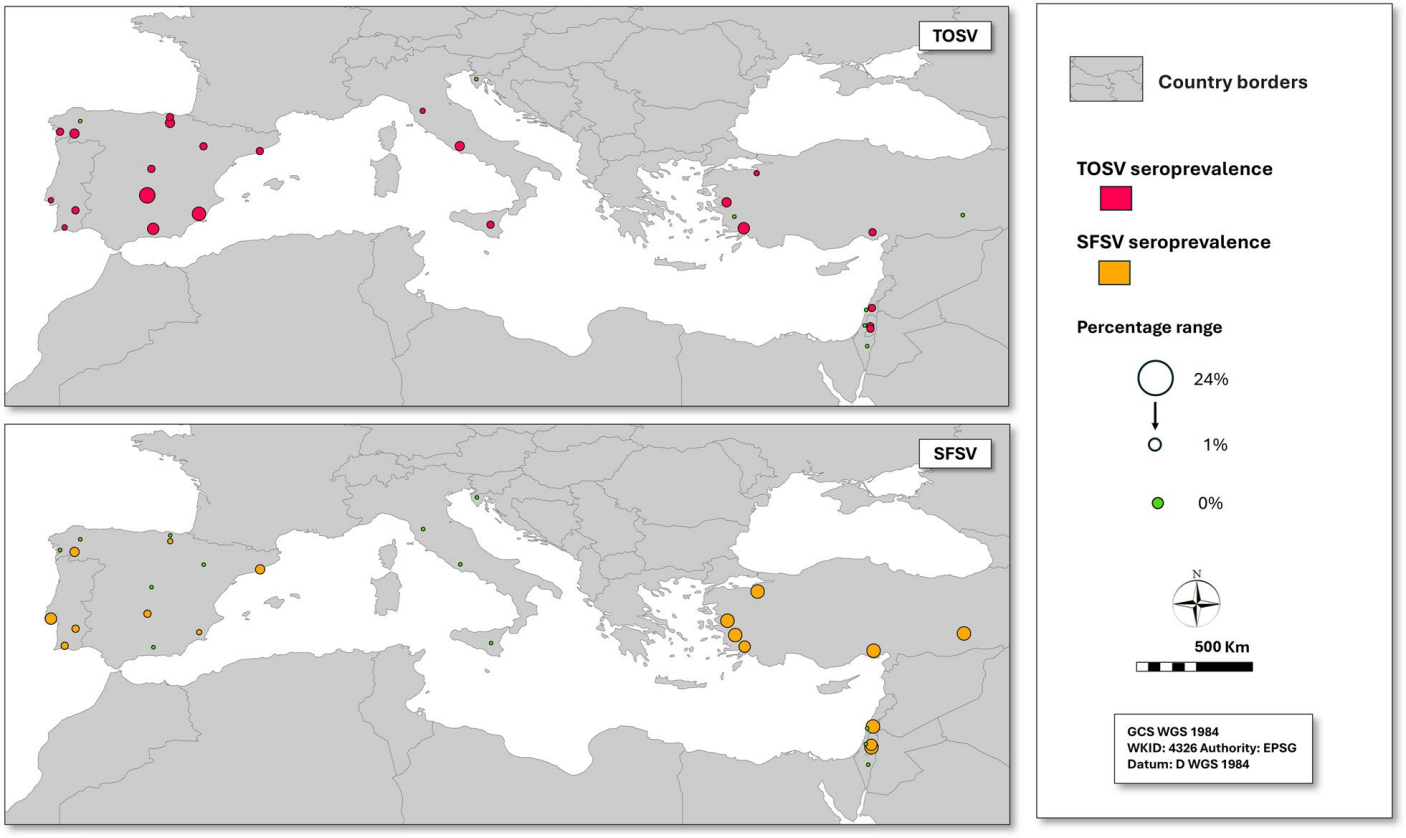
Toscana sand fly virus (TOSV) and sand fly fever Sicilian virus (SFSV) seroneutralization antibody seroprevalence in dogs in selected areas of Portugal, Spain, Italy, Croatia, Turkey, and Israel. Values are presented on a continuous scale; the largest and smallest circles correspond to the maximum and minimum values shown in the legend

Antibodies against TOSV and SFSV were detected in 110 of 2204 dogs and 103 of 2218 dogs, respectively, corresponding to estimated seroprevalences of 5% (4–6%) for both infections (Table 1). Dogs seropositive to one or both viruses were detected in all countries except Croatia, and this showed pronounced spatial clustering with 74 of the TOSV-positive dogs from Spain (9% seroprevalence), primarily from the provinces of Ciudad Real and Murcia (Table 1; Fig. 2). Likewise, SFSV infections were most common in Israel (12%) and Turkey (12%) and in Portugal (4%) compared with Spain (1%), and no SFSV positives were detected in Italy and Croatia (Table 1; Fig. 2).

On bivariate analysis, TOSV seroprevalence was particularly high in dogs from farms, in those sampled in October–December. It increased with age, and it was lower in mixed compared with pure-breed dogs and in dogs exposed to sand flies at night. In contrast, SFSV seroprevalence was highest in shelter dogs, those sampled in January-March, in mixed-breed and short-haired dogs, as well as in those exposed to sand flies at night (*P* < 0.05) (Table 1). Antibody titers to TOSV and SFSV were not associated with explanatory variables except that the proportion of high TOSV titers was lower in pure-bred compared with other dogs.

In the final mixed-effects logistic model, TOSV seropositivity was significantly positively associated with age (*P* < 0.05) and was marginally higher in autochthonous pure-bred dogs compared with other dogs (*P* < 0.10) (Table 2). SFSV seroprevalence was marginally lower in hunting kennel dogs compared with shelter dogs (*P* < 0.10) (Table 2). However, as for CanL seroprevalence, most of the dog’s TOSV and SFSV seropositivity was attributable to unaccounted differences between geographical areas, as reflected by high adjusted and unadjusted ICC (TOSV: 0.22 and 0.21; SFSV: 0.58 and 0.53) and *R*^2^ values (TOSV: 0.24 and 0.04; SFSV: 0.58 and 0.05).

### Seroprevalence of antibodies against *Phlebotomus perniciosus *and *Phlebotomus papatasi* salivary proteins and relationships with explanatory variables

The median antibody level of detection (LOD) for *P. perniciosus* PER-rSP03B was 24 (range: 0–19). On the basis of the established LOD cutoff, 1298 of 1540 dogs were classified as seropositive, corresponding to an estimated seroprevalence of 84% (95% CI: 82–86%). Similarly, the median LOD for *P. papatasi* PAP-rSP36 was 13 (range: 0–71), and 395 of 1087 dogs were seropositive, yielding an estimated seroprevalence of 36% (95% CI: 33–39%) (Table 3).

**Fig. 3:**
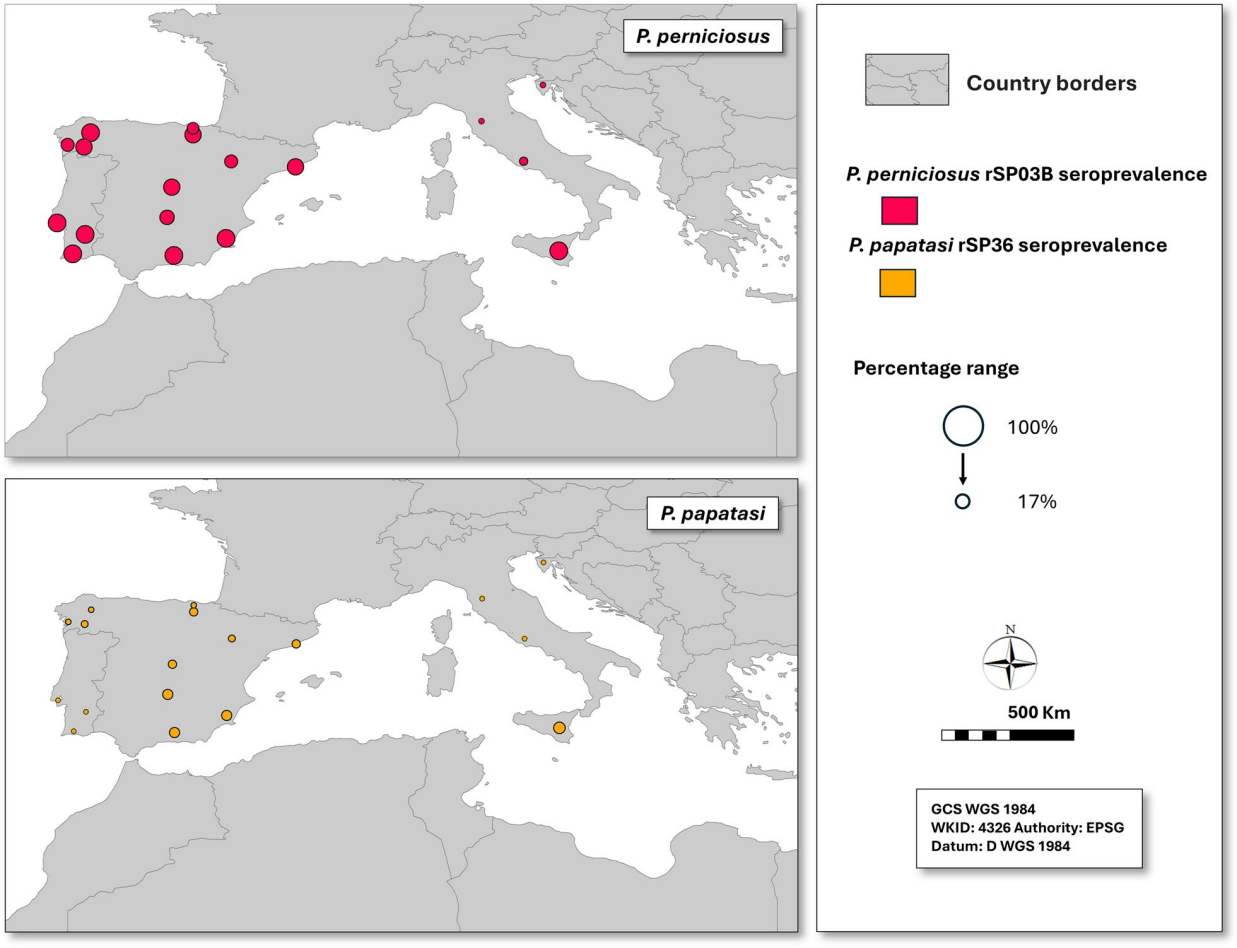
ELISA antibody seroprevalence to *Phlebotomus perniciosus* PER-rSP03B and *Phlebotomus papatasi* PAP-rSP36 salivary recombinant proteins in selected areas of Italy, Spain, and Portugal (for the latter, data are available for *P. perniciosus* only). Values are presented on a continuous scale; the largest and smallest circles correspond to the maximum and minimum values shown in the legend

However, seroprevalence varied across countries and regions. For *P. perniciosus*, seroprevalence was 100% in dogs from Portugal, 82% in dogs from Spain (ranging from 67% in Pontevedra to 95% in Granada and Lugo), and 68% in dogs from Italy (96% in Sicily, 37% in Lazio and 0% in Tuscany) (Table 3; Fig. 3). Median LODs among seropositive dogs also differed significantly, being highest in dogs from Sicily (53), followed by those from Alentejo (42) and Setubal in Lisbon M.R. (37) (*P* < 0.05) (Table 3).

For *P. papatasi*, seroprevalence was 57% in Italian dogs (tested only in Sicily) and 34% in Spanish dogs (ranging from 17% in Lugo and 18% in Bizkaia to 41–43% in Ciudad Real, Murcia and Granada) (*P* < 0.05) (Table 3; Fig. 3). Median SFSV LODs among seropositive dogs were similar across regions (Table 3).

Bivariate analysis of *P. perniciosus* and *P. papatasi* salivary antibody seropositivity with other explanatory variables indicated that seroprevalence was highest in shelter dogs (numerically only), in those sampled in 2024–25, in dogs aged 3.1–7.0 years, and in those sleeping outdoors (marginally for *P. perniciosus*), and lowest in dogs treated simultaneously with sand fly repellent-insecticide pipettes and collars. Also, *P. perniciosus* seroprevalence was lowest in dogs who did not use ectoparasiticides and with no clinical signs of leishmaniosis (*P* < 0.05) (Table 3). Among seropositive dogs, median LODs differed according to several dog-level variables and were positively associated with males, mixed breeds, short haired, hunting kennels, high night exposure to sand flies, using ectoparasiticides and specifically for sand flies, not using leishmania vaccines and not having clinical signs (Table 3).

The mixed-effects logistic regression model of *P. perniciosus* PER-rSP03B seropositivity indicated marked spatial heterogeneity (adjusted ICC = 0.527; unadjusted ICC = 0.479; marginal *R*^2^ = 0.09; conditional *R*^2^ = 0.57). Although fixed effects explained only a limited proportion of the total variability, seropositivity was significantly higher in dogs housed in shelter kennels compared with other dogs, whereas the use of sand fly-specific repellents was associated with a reduced probability of seropositivity (Table 2).

In the *P. papatasi* PAP-rSP36 mixed-effects logistic regression model, spatial clustering was comparatively small, though not negligible (adjusted ICC = 0.061; unadjusted ICC = 0.059; marginal *R*^2^ = 0.03; conditional *R*^2^ = 0.09). Furthermore, seropositivity was significantly greater in dogs with high night exposure to sand flies and lowest in the youngest age group (Table 2).

## Discussion

This study provides a comprehensive analysis of the seroprevalence of *L. infantum*, TOSV, and SFSV, along with associated risk factors, in dogs across a broad latitudinal gradient in Mediterranean European countries, Turkey, and Israel, as well as an assessment of canine exposure to major sand fly vectors *P. perniciosus* and *P. papatasi* in the Iberian Peninsula and Italy. Antibodies against *Leishmania* and phleboviruses as well as vector exposure were widely detected, although seroprevalence varied considerably between regions.

### Canine leishmaniosis seroprevalence

No *Leishmania*-seropositive dogs were detected in the Basque Country (Bizkaia and Araba) in northern Spain, in Croatian Istria, or in several districts of Israel, where overall seroprevalence was generally very low. In contrast, *Leishmania* seroprevalence ranged from 31% to 54% in Sicily, in the Turkish provinces of Aydin, Adana and Manisa, and in Ciudad Real in Spain.

The estimated CanL seroprevalence in Spain in the present study was 15%, exceeding the national prevalence of 10% reported a decade earlier in a nationwide review [38]. The geographical distribution of seroprevalence showed partial agreement between the two studies, particularly in Barcelona, the Basque Country, Galicia (Lugo, Ourense, and Pontevedra), and Zaragoza. In contrast, substantially higher seroprevalence was observed in Ciudad Real, Granada, and Madrid compared with previous estimates [38, 39]. Seroprevalence in Murcia was also higher than values reported in earlier regional studies [40–42].

In Portugal, the estimated seroprevalence was 9%, which is lower than the 13% reported in a nationwide survey conducted in 2021 [43]. This difference was even more pronounced at the regional level, with seroprevalence in Algarve and Alentejo being less than half of previously reported values, and a lower prevalence in Setúbal in Lisbon M.R. (11% versus 16%).

A previous study conducted in Sicily described a similarly high CanL seroprevalence, with the Island of Lampedusa showing a prevalence of 54% [44]. Remarkably, in a study carried out in Lazio involving 13,292 serum samples from kennel and owned dogs, an overall true seroprevalence of 7% (95% CI: 6–7%) was estimated for the entire population [45], whereas in the present study the seroprevalence in the same area was 18%. Canine leishmaniosis is also endemic in Tuscany, with early serological surveys in the coastal focus of Monte Argentario (Grosseto) reporting canine seroprevalence of approximately 24% with a proportion of dogs exhibiting clinical disease [46, 47].

In Croatia, endemic transmission of canine leishmaniosis has been historically documented in central and southern coastal and insular regions, whereas the northern Adriatic has consistently remained outside the recognized endemic zone [48–50]. Accordingly, all seropositive dogs identified during the study period were epidemiologically linked to southern Croatian coastal areas, supporting the absence of autochthonous transmission in Istria.

Canine leishmaniosis in Turkey shows pronounced geographical heterogeneity. Previous studies, largely conducted in endemic areas with reported human and canine cases, estimate a nationwide seroprevalence of approximately 15%, with values ranging from 0% to 27% and higher prevalence in areas reporting human cases [51]. Substantial local variation has been documented even between neighboring villages, with seroprevalence ranging from 0% to 23% depending on the presence of human cases [52]. In line with these findings, the present study identified marked regional differences, with seroprevalence ranging from 10% in Diyarbakır to 54% in Aydın, likely reflecting the diverse geographical and climatic conditions across the country.

A recent retrospective study of canine leishmaniosis in Israel indicated that infection was mostly found in central and northern Israel [53]. The results of the current study confirm the presence of *L. infantum* infection in northern Israel (2%) and the Haifa region (8%) which is also in the north part of the country; however, infection rates were low compared with those found in countries of the northern Mediterranean coast and Portugal. Canine infection with *Leishmania tropica* and *L. major* has also been reported from Israel; nonetheless, the prevalence of clinical disease caused by these two species in dogs is far lower than that of *L. infantum* [53].

Discrepancies between studies in the same areas must be interpreted cautiously, as seroprevalence estimates are strongly influenced by differences in the sampled populations and the diagnostic methods employed. Considerable variability exists within the most common serological assays for canine leishmaniosis, IFAT and ELISA. For instance, IFAT cutoffs defining seropositivity were set at 1:100 in the cross-sectional survey by Gálvez et al. [38] and at 1:160 in the study by Martín-Sánchez et al. [39], compared with 1:80 in the present study. ELISA performance can also vary widely [40, 54, 55], largely depending on the antigen used, such as crude promastigote preparations versus recombinant proteins [55]. In the national serological survey in Portugal, Almeida et al. [43] assessed the dog’s serological status using the direct agglutination test (DAT) and in some field studies IFAT sensitivity was lower compared with DAT [56].

Seroprevalence provides an estimate of infection prevalence and, by extension, transmission risk. In vector-borne diseases, this risk is influenced by vector infection rates as well as factors affecting dogs’ exposure to sand flies and their susceptibility to infection. In the present study, mixed-effects logistic regression analyses indicated that seropositivity to *Leishmania*, as well as to phleboviruses and *P. perniciosus* salivary antigens, was largely determined by geographical context, with most of the explained variance attributable to differences between regions rather than individual dog-level factors. This pattern reflects marked spatial clustering of transmission, likely driven by unmeasured locality-level factors such as environmental conditions affecting vector abundance and infection rates, reservoir host density, and overall transmission intensity. Some of the variation between countries may be explained by differences in sampling periods, which were not temporally synchronized across study sites. Moreover, although countries such as Turkey and Israel share certain ecological and epidemiological characteristics with Southern Europe and are part of the wider Mediterranean endemic zone, they have more diverse vector species composition, reservoir systems, environmental conditions, and surveillance settings [28, 57], which may contribute to additional variability in the observed patterns. Ongoing analyses of sand fly infection rates within the CLIMOS project are expected to further clarify these spatial dynamics. Dog-level risk factor analyses are likely to be most informative in areas with moderate to high transmission intensity.

Despite the dominant role of geographical heterogeneity, the model identified several significant dog-level predictors of *Leishmania* seropositivity. As expected from the epidemiology of CanL, positive associations were observed between seropositivity and behaviors that increase vector exposure, such as sleeping outdoors or in open-air structures [39], as well as the presence of clinical signs compatible with canine leishmaniasis. Conversely, the use of preventive ectoparasiticide treatments was negatively associated with *Leishmania* seropositivity, supporting their protective role. In contrast, the use of specific sand fly repellent products and CanL vaccination were positively associated. However, these associations should not be interpreted as evidence of a detrimental effect of repellents or vaccination. Rather, they are most likely explained by confounding related to owner behavior and spatial setting. Dog owners living in areas with higher transmission risk may be more likely to apply sand fly repellents and to vaccinate their animals than owners in lower-risk areas, leading to an apparent positive association in observational data. Additional factors may also contribute to the latter findings. LetiFend is a differentiating infected from vaccinated animals (DIVA) vaccine designed so that vaccinated dogs do not test positive on standard serological assays such as IFAT [58]. However, while the vaccine reduces the incidence of clinical leishmaniosis, it does not necessarily prevent infection or onward transmission of *Leishmania* [59, 60]. Consequently, vaccinated dogs may still become naturally infected and seroconvert. Similarly, the efficacy of sand fly repellents is below 100% and can be further reduced by practical factors such as owner noncompliance with recommended application schedules, spot-on formulations being washed off before absorption, dogs being bathed or swimming shortly after treatment, or incorrect application techniques [41, 43].

### TOSV and SFSV seroprevalence

Toscana virus (TOSV) seropositivity was absent in dogs from Istria and from several regions of Israel, Turkey, and Spain, and remained low in most other areas, except for Ciudad Real (24%) and Murcia (16%) in Spain, and Mugla (10%) in Turkey. Sand fly fever Sicilian virus (SFSV) seroprevalence was highly focalized, occurring mainly in Turkey (12%) and in several districts of Israel (7–15%) and Setúbal in Lisbon M.R. (7%), while it was absent from Istria, Italy, and many parts of Spain.

Only one previous serological survey of TOSV in dogs has been conducted in Spain, in Granada during 2006–2007, reporting an IFAT seroprevalence of 48% [61]. This figure is substantially higher than the 8% seroprevalence observed in dogs from the same region in the present study using seroneutralization (SN). This discrepancy is likely due in part to the higher specificity of the SN assay—the gold standard for phlebovirus serodiagnosis—which reduces false-positive results [62], as well as to differences in TOSV exposure among the dog populations sampled, despite originating from the same province. Evidence from studies in humans and other animal species indicates that TOSV circulates widely in Spain, although reported seroprevalence varies markedly according to host species and geographic area. In Murcia, Ortuño et al. [63] reported a TOSV SN seroprevalence of 26% in human blood donors, a figure broadly comparable to the 16% seroprevalence observed in dogs in the present study. In Madrid, Martínez et al. [64] estimated TOSV SN seroprevalence in humans of 42% in samples collected in 2007, and 27% in samples from 2018 to 2019, both substantially higher than the 3% seroprevalence found in dogs in the current study. These human seroprevalence levels are also consistent with the high exposure of phleboviruses recently reported in wildlife in Spain, including seroprevalence rates above 40% for both TOSV and SFSV in wild common quails (*Coturnix coturnix*) in northeastern Spain [65], and 23% for TOSV and 10% for SFSV in European bats in southern Spain [66]. In contrast, the SFSV seroprevalence in dogs in the present study was very low throughout the country. Although no previous serological surveys for SFSV in dogs have been reported in Spain, human data suggest limited circulation of this virus in the domestic environment: Ortuño et al. [63] found 0% SFSV seroprevalence among blood donors in Murcia, consistent with earlier studies reporting minimal or undetectable levels of SFSV antibodies in general human populations in Spain.

In Portugal, the TOSV positivity rate (1%, 0–6%) was lower than that reported in a previous study of dogs in the south of the country (6%, 5–7%); in that study, TOSV prevalence in the Algarve (13%) was significantly higher than in Lisboa (7%) and Setúbal (5%) [67]. In humans, a total of 12 TOSV cases have been reported in Portugal to date [11], with the first documented case dating back to 1983, demonstrating the longstanding circulation of the virus in the country. Seroprevalence studies conducted using different diagnostic techniques in the districts of Porto, Lisbon, Santarém, and Setúbal reported rates ranging from 1% to 5% among healthy blood donors. In 2022, the estimated national TOSV SN seroprevalence was 3% (95% CI: 2–3%). Significative regional variation was observed, with the highest seroprevalence values—reaching 15%—in southern and northeastern parts of the country. In contrast, seroprevalence in the Algarve region was 2%, closely aligning with the 1% observed in dogs in the present study, while Setúbal and Alto Alentejo showed a prevalence of 4% and 15%, respectively [68]. Regarding SFSV in Portuguese dogs, although seroprevalence in dogs in the present study was higher (4%) than that observed for TOSV (1%), it was considerably lower than the levels reported by Alwassouf et al. [67], who documented seroprevalence rates of up to 51% in dogs from central and southern regions of the country and 55% in Setúbal—significantly higher than in the present study. These discrepancies between studies may be partly explained by differences in the SN positivity thresholds applied: the earlier study considered a neutralization titer of ≥ 20 as positive, whereas the present study used a more stringent cutoff of ≥ 40. To date, only one human case of SFSV has been reported in Portugal, occurring in a child [69]. A regional seroprevalence study conducted in healthy individuals from the Peninsula de Setubal region reported a value of 4%, of which the estimated national true seroprevalence in blood donors in 2022 was 5% (95% CI 3–6%). Significant differences in regional seroprevalence were found, with the highest values (up to 12%) in Algarve, Alentejo, and Lisbon M.R. [68].

Italy is currently the only country with active surveillance for TOSV in humans during the warm season. The circulation of TOSV is well documented, and human cases are reported by public health authorities, with annual case numbers potentially reaching up to 100, including some fatal cases [70]. Seroprevalence studies in humans have reported seropositivity rates of 33% for TOSV IgG and 9% for SFSV in Sicily [31]. In our study, the dog seroprevalence rates were low for TOSV (3%) and no seropositivity was detected against SFSV, which can be explained by testing method differences.

In Turkey, a total of 31 TOSV human cases were reported since 2010, with symptomatic infections observed in immunocompromised individuals and in young adults [31]. TOSV was also implicated as a potential vector-borne trigger for Guillain–Barré syndrome in a case–control study [71]. Additionally, outbreaks caused by SFSV, locally circulating SFSV clade, sand fly fever Turkey virus (SFTV) have been documented in Ankara [71]. A recent seroprevalence study in blood donors revealed that 22.6% had been exposed to TOSV, while 12.1% showed evidence of exposure to SFSV in Central Anatolia [72, 73, 74]. In our study, the seroprevalence rates in dog sera were 4% for TOSV and 12% for SFSV. These differences may be attributed to variations in collection sites, sampling periods, and differential exposure between humans and dogs.

Although Toscana virus (TOSV) infections have been reported in human patients and detected in sand flies in Croatia, and seroprevalence studies have indicated high levels of exposure—particularly on the Croatian islands [75–78], the absence of TOSV and SFSV seropositivity among dogs in the present study is likely due to sampling bias, as only 27 dogs were sampled at a single location in the Istria region.

Limited information is available on sand-fly-borne phleboviruses in Israel. To date, only three human cases have been reported in the literature [79, 80], and to the best of our knowledge, this is the first seroprevalence study conducted in the country. Our results showed serological reactivity compatible with SFSV (12%) and a comparatively lower circulation of TOSV (2%) in dogs. However, given the limited published data on phleboviruses in Israel and the possible serological cross-reactivity among closely related phleboviruses, these findings should be interpreted with caution and may reflect exposure to antigenically related, yet undescribed phleboviruses circulating in the region rather than true SFSV circulation.

### Seroprevalence to *Phlebotomus perniciosus* and *Phlebotomus papatasi* salivary antigens

Seropositivity to *P. perniciosus* saliva was very high (80–100%) in Portugal, Sicily, and most regions of Spain, including northern areas, but was lower in Lazio (37%) and absent in Tuscany. Likewise, *P. papatasi* seroprevalence reached 57% in Sicily and ranged from 17% to 43% in Spain. Geographical variations in seroprevalence to *P. perniciosus* and *P. papatasi* salivary antigens broadly reflect the known distribution of these sand fly species [28]. *Phlebotomus perniciosus* is the principal vector and the most abundant species in the Iberian Peninsula, and is also commonly found in Sicily, Lazio, and Tuscany [81–84]. In contrast, *P. papatasi* is widespread in Spain, albeit at low density, and occurs less frequently in Mainland Italy and Sicily [83].

The *P. perniciosus* seropositivity observed in Portuguese dogs aligns with previous findings from the Lisbon M.R., where Maia et al. [85] reported 86% at the beginning and 95% at the end of the sand fly season. The high seroprevalence to *P. perniciosus*, and to a lesser extent, to *P. papatasi* salivary antigens, in the northern Basque provinces is unexpected. Sand fly abundance in this region is low, and the only species reported so far are *Phlebotomus ariasi* and *Phlebotomus mascittii*, which are adapted to cooler and more humid climates [86, 87]. While it seems unlikely that most dogs had traveled to areas endemic for *P. perniciosus*, limited or sporadic travel, particularly among dogs from Araba, cannot be excluded as a potential contributing factor. These findings may reflect serological cross-reactivity with antigens from other sand fly species, particularly those in the same subgenus. This was investigated for PER-rSP03B using sera from mice experimentally exposed to a single species [88]. In western blot, it was species-specific against anti-*P. papatasi* antibodies but cross-reacted with anti-*Phlebotomus tobbi* antibodies, a species in the same subgenus as *P. perniciosus*. Recently developed *P. tobbi* salivary recombinant proteins also cross-reacted with murine anti-*P. perniciosus* antibodies in ELISA. Anti-*P. ariasi* antibodies were not tested, but cross-reactivity with other *Larroussius* species cannot be excluded in mice or dogs. We therefore cannot interpret these serological results as definitive evidence of local vector exposure without entomological confirmation. Nevertheless, because both *P. perniciosus* and *P. ariasi* are proven *L. infantum* vectors, such cross-reactivity may still yield epidemiologically meaningful information.

The modeling analyses identified significant and epidemiologically consistent associations between seropositivity to *P. perniciosus* and *P. papatasi* and several dog-level factors. Higher seropositivity was observed in dogs kept in shelter kennels (for *P. perniciosus*), which are generally open-air environments, in dogs that spend the night outdoors (for *P. papatasi*), and in older dogs (for *P. papatasi*). Conversely, seropositivity was lower in those receiving sand fly repellent treatments (for *P. perniciosus*). In Mediterranean regions, adult sand fly activity typically begins at dusk and peaks between 11 pm and 3 am [81], placing outdoor dogs at substantially higher risk during the night. The age-related association likely reflects cumulative exposure over time, with older dogs producing higher levels of antisaliva antibodies. Detection of antibodies in dogs treated with repellent insecticides, although at a lower frequency than in untreated ones, was not unexpected. As noted above, these products do not provide complete protection, and their effectiveness may be further reduced when manufacturer recommendations are not strictly followed [41].

## Conclusions

By using dogs as a sentinel species, this study demonstrates, through a single standardized sampling and diagnostic framework, marked heterogeneity in sand-fly-borne infections across a broad Mediterranean endemic region and identifies the main drivers of this variability. Regional patterns of *Leishmania* and phlebovirus infections were broadly consistent with previous reports, although seroprevalence in some areas differed. These results underscore fine-scale variability in infection risk and the importance of accounting for it when developing risk maps and control strategies. Differences between regions are likely driven primarily by variation in the density of infected sand fly populations. Entomological data from the CLIMOS project will be incorporated in future analyses to further investigate these patterns. In this framework, canine serological data provide an independent biological layer to support calibration and validation of spatial risk models. Together, these data will inform predictive models and support geographically targeted interventions, which combined with evidence-based dog-level preventive measures, are essential for effective control of sand-fly-borne infections.

## Data Availability

The data supporting the conclusions of this article are included in the tables within the article.
